# Transactions of the New Hampshire Medical Society, 1859

**Published:** 1859-12

**Authors:** 


*Transactions of the New Hampshire Medical Society*, 1859.

The report of the Committee on Surgery, by W. D. Buck, M.D., contains several interesting points of practice. Among these we notice a suggestion to place the long splint on the outside of the sound thigh, in fractures of that part, when the extension or counter extension has produced excoriation of the perineum, or on the ankle on the side of the fracture. Dr. Buck recommends the use of adhesive plaster for extension in these fractures, attributing its first use in this way to Dr. Josiah Crosby, of Manchester, N. H., who, he says, is called "Sticking Plaster Crosby." We hope the name may adhere.

The following passage on amputation at the shoulder joint is interesting. The suggestion to remove the head of the humerus in order to facilitate the tieing of the artery, we do not remember to have seen elsewhere.

*Amputation of Shoulder Joint, by Josiah Crosby, M.D.*—Within the last fourteen years I have amputated the shoulder joint four times, and removed the head of the humerus once. The first case was gun-shot wound; the second and third railroad accidents; in the fourth the arm was caught in the gearing of a flouring mill. In all these cases except the last, the bones were crushed to the head; in the last, the one caught in the mill, the bone was uninjured high up, but the brachial artery was completely severed high up. The integuments in three of the cases were so destroyed that it was impossible to get clear cut edges for dressing. In all of the operations the head of the bone was disarticulated and removed from its socket first, then the artery was easily reached and secured. The soft parts were then trimmed with as much economy as possible, to give covering to the wound. The first three cases made a good recovery. In the fourth case, an attempt was made to save the arm, but on the second day there was manifestly such a want of vitality that the operation was performed, but with no advantage to the patient, who died on the fourth day from the injury.

*Perforation of the Femur, by Josiah Crosby, M.D.*—Mr. H., aged thirty-five, when ten years old, had necrosis of the lower end of the femur, near the external condyle. During the progress of the disease, he fell and fractured the bone at the place affected. Both recovered together, and he had apparently a good limb, until March, 1857, when he was attacked with pain in the seat of the old disease. The pain intermitted, so that for days he would be perfectly free from it, when it would return, increasing in severity and duration, until he was obliged to relinquish his business (a machinist) and devote himself entirely to the care of his limb. The pain became so severe that he was obliged to resort to large quantities of morphine and ether to get any rest. His flesh wasted; the whole limb was much smaller than the other; no swelling and but little tenderness, and that only when considerable pressure was made on the seat of the pain. A great variety of treatment was resorted to with no permanent benefit, and with very little temporary relief. Several surgeons were consulted, but all to no purpose; the patient constantly grew worse until he and his friends were fearful that he might lose limb or his life.

I visited him on the fourteenth of January, 1858, and proposed perforation of the bone, Dr. Hubbard, of this city, and Dr. Paige, of Candia, his attending physician, being present. The operation was performed the same day. An incision one inch long was made on the outside of the leg, about three inches above the articulating surface of the external condyle, and, at the suggestion of my friend Dr. Buck, the perforation was made nearly through the cancellated structure, with a three-eighths of an inch gouge bit, placed in a common bit-stock. The instrument perforated the bone with perfect ease in a half a minute. The bone appeared perfectly healthy, and no pus appeared. The wound was kept open by lint to prevent immediate union. The pain ceased a few days after the operation was performed, and the patient's appetite soon returning, he rapidly recovered, and is now enjoying good health with apparently a sound limb.

We had suggested the sub-cutaneous perforation of bone many years since, with the instrument in use for ununited fracture, but have not found occasion to put it in practice. In commencing abscess, if used early, it would seem to promise much.

